# Basic ctDNA Panel Promises Affordable Clinical Validity in Colon Cancer Patients but Not in Pancreas Cancer Patients

**DOI:** 10.3390/life13122274

**Published:** 2023-11-28

**Authors:** Mandy Radefeldt, Silke Stellmacher-Kaiser, Susann Krake, Brigitte Kragl, Sabrina Lemke, Christian Beetz, Peter Bauer, Christian Junghanß, Ruslan Al-Ali

**Affiliations:** 1 CENTOGENE GmbH, Am Strande 7, 18055 Rostock, Germanysusann.krake@centogene.com (S.K.);; 2 Clinical for Internal Medicine, Hematology, Oncology and Palliative Medicine, University Medicine Rostock, Ernst-Heydemann-Str. 6, 18057 Rostock, Germany

**Keywords:** ctDNA, cancer, colon, pancreas, panel, biomarker

## Abstract

The potential of circulating tumor DNA (ctDNA) as a biomarker to assess the progression of various solid tumors has been explored extensively. In this study, we investigated the feasibility of utilizing a ctDNA sequencing panel specifically designed to target the most frequently mutated genomic regions in colon and pancreas cancers. Through somatic analysis of colon and pancreas tumors, we targeted 27 regions within eight genes. By employing PCR amplification and Illumina NGS, we ensured that each region was adequately covered with a minimum of 5000 reads (with an average of 12,000 reads). Our method exhibited reproducibility with repetition and dilutions. The positive detection threshold for ctDNA was set at a cutoff value of 0.5% ctDNA of the total reads using IGV. Among the samples analyzed, 71% of colon cancer cases displayed somatic mutations covered by the targeted regions. Within this group, detectable ctDNA was observed in 34% of the cases. Conversely, in pancreatic cancer, 55% of mutations were covered by the panel’s regions, but only 13% of these cases exhibited detectable ctDNA. In follow-ups with the patients, changes in ctDNA percentages demonstrated complete concordance with changes in the clinical condition in 88% of the cases. Our findings suggest that employing a basic ctDNA-targeted panel can serve as a cost-effective and reliable approach for repeated monitoring of the efficacy of colon cancer therapy. However, in the case of pancreatic cancer, ctDNA showed limited utility, and alternative biomarkers may offer superior diagnostic value. Additionally, we found that a negative ctDNA test is not a guarantee for a relapse-free recovery; thus, we recommend a continuous follow-up with the patient on a long-term basis.

## 1. Introduction

The quest for effective methods to monitor the development, recurrence, and metastasis of tumors has been a longstanding challenge in the field of oncology. Responses to various treatment modalities, including surgery, radiation therapy, and chemotherapy, can vary significantly from one patient to another. Over the years, numerous techniques and tools have been deployed to measure and predict the progression of cancer cases. These encompass cutting-edge imaging technologies and the identification of biomarkers. Our involvement in the Peptide-based Immunization for Colon- and Pancreas-Carcinoma consortium (PiCOP) project raised the need for methods to assess the efficacy of immune treatments. In addition to traditional imaging and antigen-based approaches, we explored alternative biomarkers, aiming for methods that are more sensitive, versatile, and cost-effective.

In 1948, the discovery of circulating cell-free DNA (ccfDNA) in various bodily fluids outside of cells marked a significant breakthrough. The pursuit of the ideal biomarker for cancer patients has led to a surge of interest in detecting and analyzing ccfDNA in liquid biopsies, offering potential benefits in early cancer detection and in monitoring treatment responses. Elevated levels of both ccfDNA and its subset, circulating tumor DNA (ctDNA), have been consistently associated with the presence of cancerous tissues [1,2,3,4,5,6]. While ccfDNA is predominantly derived from hematopoietic cells [7], ctDNA originates from tumor growth and is released into the bloodstream, often correlating with the stage of the disease [8]. This correlation allows us to utilize ctDNA to estimate the effectiveness of therapy and to predict tumor relapse and metastasis by monitoring the ctDNA load [9].

ctDNA typically constitutes a mere 0.5% of the total ccfDNA [10], making precise detection methods essential. With the advent of affordable next-generation sequencing (NGS) technologies, several methods have been developed to leverage the power of this sequencing approach. The cost of a ctDNA test is estimated to be half the cost of any other monitoring method [11]. One such approach is the tumor-informed strategy, exemplified by Signatera (Natera Inc., Austin, TX, USA), which involves identifying somatic mutations in tumor tissue postsurgery and designing a panel of targets to detect these mutations within ccfDNA, subsequently estimating their portion as ctDNA. Another approach involves using plasma-only-based tumor-agnostic minimal residual disease (MRD) assays, such as GuardantReveal (Guardant Health, Palo Alto, CA, USA). However, it is important to acknowledge that these methods are proprietary and often come with significant costs, especially when employed on a large scale.

Our objective was to produce an open-source panel for studying ctDNA in different types of tumors. The panel’s concept relies on targeting frequently mutated regions via basic amplification and then sequencing with NGS. The plan is to find a balance between efficacy and the cost of such a panel. We applied such a panel in participants with colon and pancreas cancers enrolled in the PiCOP project. The primary goal of the project is to study the tumors, establish peptide-based immune therapies, and study the efficacy of these and other therapies. To evaluate the efficacy of the therapy, identifying reliable biomarkers is critical. ctDNA emerged as an ideal candidate due to its high specificity and sensitivity. Additionally, a rapid clearance time and a short half-life ranging from 16 min to 2.5 h is essential, too [12].

The selection of the regions was based on a parallel sequencing of both tumor and germline DNA from blood samples. With prior knowledge of tumor-specific variants, we investigated various methodologies to measure ctDNA. Ultimately, we reached the conclusion that a panel covering frequently mutated regions can provide a well-balanced approach for tracking tumor progression and assessing treatment outcomes. The open nature of such a panel makes it easier and cheaper to modify or copy to be used in other types of cancers or other populations. 

## 2. Materials and Methods

### 2.1. Ethical Approval

All samples were collected within the “Peptide-based Immunization for Colon- and Pancreas-Carcinoma” (PiCoP-Global) initiative. This study was approved by the Ethics Committee at the Medical Faculty of the University of Rostock: approval A 2019-0026, 25 February 2019. This study was also registered in ClinicalTrials.gov with the number NCT03871790.

### 2.2. Samples Desciption

A total of 116 participants were included in this ctDNA study, comprising 86 individuals diagnosed with colon cancer and 30 individuals diagnosed with pancreatic cancer. Subsequent to the surgical removal of the primary tumor, all patients underwent curative treatment. Blood samples, tumor tissue specimens, and ccfDNA were collected from these participants. The ccfDNA was initially obtained during the primary tumor surgery, with some patients undergoing additional follow-up samplings to assess the presence of ctDNA in both colon and pancreatic samples.

Within 24 h of sample collection, ccfDNA BCT Tubes (Streck, La Vista, NE, USA) were subjected to double centrifugation, and the resulting plasma was preserved in Falcon tubes at −20 °C. Extraction of ccfDNA from the plasma was performed using a QIAamp minElute ccfDNA Kit (QIAgen, Hilden, Germany). Following extraction, the ccfDNA samples were stored at −20 °C for subsequent analysis.

### 2.3. Somatic Analysis of Primary Tumor

Whole exome sequencing (WES) was performed for all patients from pairs of blood and tumor samples. Enrichment was performed using double-stranded DNA capture probes against the exon regions of the genome (Twist Human Core Exome Plus kit, Twist Bioscience, South San Francisco, CA, USA). The generated libraries were sequenced on the Illumina NovaSeq6000 platform using the 150 paired-end protocol. The data were demultiplexed using Illumina’s bcl2fastq tool, and samples were aligned with BWA-MEM [13] against hg19. Somatic variants were called with Strelka2 [14]. Variants were annotated using snpEff v5.0 [15]. Somatic variant allele frequency was set to minimum 0.1 and a coverage of over 100 reads.

### 2.4. The Development of the ctDNA Panel

Somatic mutations were used to identify the portion of ctDNA in the total ccfDNA. The attempts to detect ctDNA in plasma, using whole exome sequencing or dedicated oncology NGS panels (CentoCancer^®^ comprehensive), failed due to relatively low coverage (20× and 200×, respectively). 

**Figure 1:**
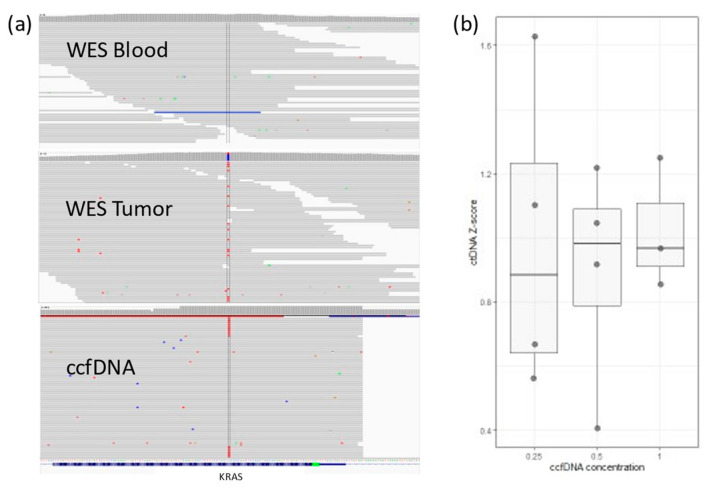
(**a**) IGV view for KRAS somatic mutation in a colon cancer patient. The mutation (in red) does not exist in WES from blood, but it can be detected in WES from tumor tissue. ctDNA sequences detected in ccfDNA (~1%). (**b**) Lower ccfDNA input correlates with higher variability in detected ctDNA concentration (Z-score is relative ctDNA expression in one sample across different concentrations).

We tested targeted PCR amplification. In 4 patients, ten personalized primer pairs per patient were designed to cover a single somatic mutation each. PCR products were sequenced using the Illumina NextSeq500 platform using the 150 paired-end protocol. Reads were aligned against hg19 using BWA-MEM. Somatic variants were inspected with Integrative Genomics Viewer-IGV (Figure 1a). A cutoff of somatic mutation ≥0.5% of the total reads (i.e., at least 0.5% of all ccfDNA is ctDNA) was set, and values between 0.1 and 0.5% were considered a gray zone.

### 2.5. Statistical Analysis

Statistical analysis for figures with boxplots was performed using the Wilcoxon test embedded in the geom_signif v.0.6.4 package [16]. The Spearman correlation test was used to evaluate the correlation between the ctDNA ratio and the various clinical data (i.e., age, age at participation, WHO clinical stage, etc.).

### 2.6. Naïve Detection of Somatic Mutations 

To evaluate the use of the panel for naïve detection of the somatic mutations, we developed a script to call variants from ctDNA BAM files using deepSNV v. 1.42.1 [17]. We did not use ccfDNA from healthy individuals due to the low amounts extracted. Rather, we used ccfDNA from tumors that had no somatic mutations within the targeted regions. This ccfDNA functioned as a “negative control”. The PCR product introduced high levels of false-positive calls in both negative controls and patients, preventing the use of this protocol without prior knowledge of the somatic mutations.

## 3. Results

### 3.1. The Development of the ctDNA Panel

**Figure 2:**
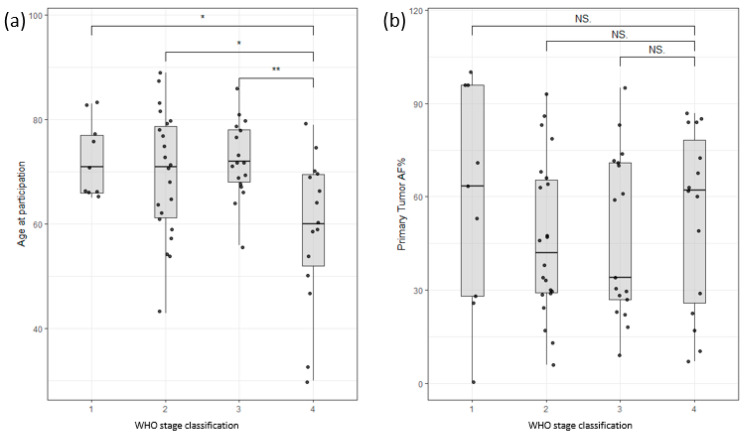
(**a**) Distribution of participants based on age of participation versus WHO stage of disease classification. Stage 4 shows a clear bias towards lower age of participation/onset. (**b**) Distribution of allele frequency of somatic variants of the primary tumor versus WHO stage of disease classification. No significant correlation could be found (Stages are grouped by major Arabic numeral, e.g., Stage 2A and Stage 2B are merged as Stage 2. ** signifies *p*-values less than 0.001, * signifies *p*-values less than 0.01, NS. stands for non-significant *p*-values, i.e., over 0.05).

The study cohort consisted of 54 male and 21 female colon cancer patients and 11 male and 12 female pancreatic cancer patients. The age range for male colon cancer patients was 30–89 years, with an average age of 70, while female colon cancer patients had an age range of 43–87 years, with an average age of 70. Interestingly, male patients with late-stage colon cancer exhibited an earlier age of onset compared to those with early-stage disease, as depicted in Figure 2a. Nevertheless, this study did not identify any significant correlations between survival and factors such as age at enrollment, stage classification, or gender (Supplementary Figure S1). Furthermore, the allele frequency of somatic mutations in the primary tumor (tumor infiltration) did not show any significant correlation with the stage of the tumor (Figure 2b). These findings highlight the complexity of establishing clear correlations among clinical data, such as tumor infiltration or survival rates.

**Figure 3:**
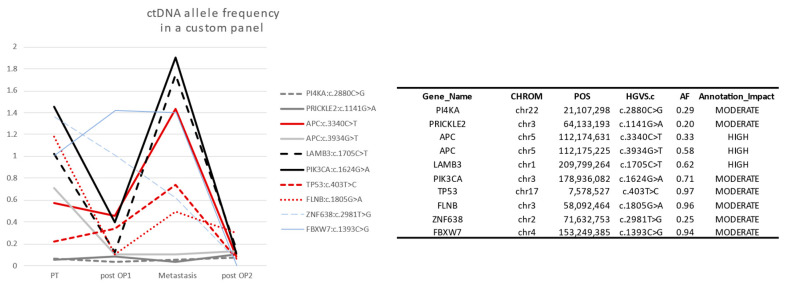
Ten selected somatic mutations detected in Sample2. Genes with low somatic allele frequency (<0.5) are not reliable to detect ctDNA (gray). The reliable genes APC, TP53, FLNB, PI4KA, and PIK3CA are high in preoperation (PT) and relapse (Metastasis), but a relative decrease happens after the first operations (post OP1), and ctDNA disappears after the second operation (post OP2). This is consistent with the clinical observations in the case study (see Section 3.4 below).

In personalized panels, ctDNA percentage followed the clinical observations in many genes. The ctDNA was higher when the tumor was growing or relapsing and lower after surgery and therapy (Figure 3). We did not see a difference in capability to detect ctDNA using single mutation versus using multiple mutation in the four patients with personalized analysis design. However, a published study stated that using one single target in ctDNA differs in preoperative comparison to using multiple targets (79% vs. 96%), but shows similar results in recurring and nonrecurring serial samples of colon cancer [18]. We noticed that somatic mutations in some regions/genes are more reliable. The mutations are repeated in those same regions for many patients. A panel covering those regions was prepared by selecting 27 regions spanning exonic sequences in the following genes: APC, TP53, KRAS, BRAF, PIK3CA, CDKN2A, SMAD4, and SALL1. The multiplex PCR was performed in two pools (Supplementary Table S1). Illumina Nextera Transposase adapter sequences were added to the forward and reverse primer sequences. The concentration of primers was optimized to obtain comparable amounts of PCR product (Supplementary Figure S2). Indexes were added to individual samples, and the samples were pooled into one mix. The pool was sequenced on the Illumina NextSeq500 platform using the 150 paired-end protocol. Reads were aligned against hg19 using BWA-MEM [13]. The variants were quantified with IGV, and the cutoff was set to a ctDNA percentage ≥ 0.5%. With a limited spike-in of concentrations, we obtain over 7000 reads covering the targeted variants. This panel is the open-source panel that is the basis of this manuscript.

### 3.2. Quality Control Parameters and Limitations

We depend in this method on detecting at least one variant in the selected regions. It was proven that using one single target in ctDNA is similar to using multiple targets in recurring and nonrecurring serial samples of colon cancer but differs in preoperative comparison (79% vs. 96%) [17].

**Figure 4:**
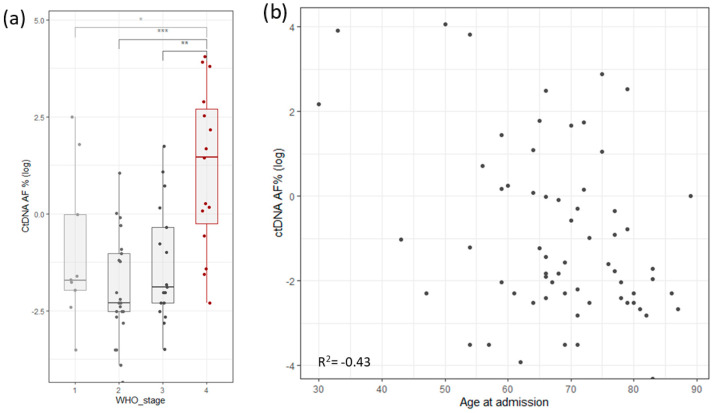
(**a**) Distribution of log ctDNA % by WHO stage of disease classification. Stage 4 shows a clear bias towards higher levels of ctDNA. Stage 1 has outliers that distort the statistics. (**b**) Correlation of ctDNA allele frequency with age of admission (Stages are grouped by major Arabic numeral, e.g., Stage 1A and Stage 1B are combined in Stage 1. *** signifies *p*-values less than 0.0001, ** signifies *p*-values less than 0.001, * signifies *p*-values less than 0.01, NS. stands for non-significant *p*-values, i.e., over 0.05).

To assess the robustness and repetitiveness of this method, we duplicated the testing of 24 samples. All positive mutations were confirmed with a cutoff of >0.5% of total ccfDNA. Sensitivity was evaluated using three dilutions for four samples. ccfDNA was diluted into 1 ng/µL, 0.5 ng/µL, and 0.25 ng/µL, measured with Qubit (Thermo Fischer, Waltham, MA, USA), and then PCR and libraries were prepared. The results based on the cutoff from different dilutions pointed to an increased variability with a lower input of ccfDNA (Figure 1b). The ctDNA percentage increased significantly in stage 4, but it also had high variability in stage 1 cancer (Figure 4a). ctDNA correlated mildly with age of admission (Figure 4b), but age of admission also correlated with stage of cancer. On the other hand, survival did not correlate with cancer stage, age of admission, primary tumor allele frequency, or ctDNA levels (Supplementary Figure S1c).

### 3.3. ctDNA Presence in Colon and Pancreas Samples

We found that 71% of colon cancer primary tumors have at least one somatic variant in the targeted regions (87 out of 122 patients). We analyzed ccfDNA from all patients with mutations covered by the targeted PCR and detected ctDNA in 30 individuals (34%). These values are within the published ctDNA levels in colon cancer that ranges between 25% and 72% (“Table 2” in [8]). Ignoring the 0.5% cutoff, the detected ctDNA value increase to 59% of the patients. However, we are suspicious of replacing quantification with detection, as decreased amounts of ccfDNA and ctDNA introduce more uncertainty and fluctuations (Figure 1b). Thus, we used the 0.5% cutoff in all samples in further analyses. 

Out of 55 pancreatic patients, 30 individuals had somatic mutations in the regions covered by the ctDNA panel (55%). We could detect ctDNA in only 4 of those 30 individuals (13%). This is comparable to the reported 18% using chip-based PCR [2]. 

We measured the levels of ctDNA using a panel to follow up the clinical development in 11 colon cancer patients and 5 pancreas cancer patients. A total of 64% of patients with colon cancer had detectable ctDNA in at least one point (7 out of 11 patients). On the other hand, only 20% pf patients with pancreas tumors had detectable ctDNA (one out of five patients). Changes in ctDNA levels correlated with the clinical changes in 88% of the cases (seven out of eight) (Table 1 and Table S2). 

### 3.4. Case Study

We obtained ccfDNA samples of five time-points from a 59-year-old male patient (Table 2). The patient had squamous cell carcinoma in the bottom of the mouth and was treated with radiation three years before his inclusion in this study.

The patient was diagnosed with colon cancer and underwent a laparoscopic sigmoid resection and regional lymphadenectomy at the first time-point. Then at the second time-point, the patient had a liver metastasis surgery and radiation. The third time-point was at surgeries for metastasis in the lungs. The fourth point yielded a negative ctDNA result 2 months after the last surgery. 

In a follow-up check, the physician suspected a relapse, but imaging could not confirm this. At this stage, ctDNA tests were positive again (fifth time-point). After 2 months, the imaging confirmed a progressive disease.

## 4. Discussion

Both ccfDNA and ctDNA have gained significant attention in the field of liquid biopsy as noninvasive methods for disease monitoring, especially cancer [5,19,20,21]. Many initiatives were set in place to study the molecular dynamics and prognostic role of ctDNA, such as CIRCULATE-US [22]. Such studies should confirm the documented correlation of high levels of ctDNA with prognostic and predictive information [23].

ctDNA is suitable for cancer studies that obtained genetic data from patients’ tumor and germline. While measuring ctDNA can be performed by droplet-based digital PCR [24], utilizing NGS targeted amplification methods is becoming more common, such as TAm-Seq, eTAm-Seq, CAPP-Seq, and TEC-Seq [25]. A somatic mutation can be detected in ccfDNA by amplifying the region around the mutation and sequencing the product. While primer design, manufacturing, calibration, and application is possible for each individual case, large-scale studies or tests in a medical center cause financial and logistical challenges. Our aim was to find the best practical approach and evaluate the cost–benefit. It is estimated that the average cost of one ctDNA test is USD 500 [11]. It was reported in the media that the Medicare program pays USD 3500 for each Signatera colon ctDNA test under initiatives to encourage pioneering genetic tests (B. Alpert, “How Natera Is Defending Its Lead in a $15B Cancer-Testing Market,” Barron’s 2021). 

Our approach is based on creating a fine-tuned panel to cover frequently mutated regions in a standardized form. Regions are selected from which to obtain unique and reliable sequencing reads. We tested replicates and different concentrations of ccfDNA, and the ctDNA was consistent in differentiating positive and negative ctDNA. Following one mutation in the panel is as effective as following a personalized panel if the somatic mutation is in the targeted reliable regions (Figure 3). Our method is semiquantitative due to using basic PCR amplification. However, it can be converted into a quantitative method using a unique molecular identifier (UMI). Naturally, this is accompanied by additional cost and complexity, and with basic PCR performing adequately, there is limited benefit. In a price-sensitive medical environment, having an economic, flexible, and sensitive method is essential to providing good medical service in both developed and developing countries. 

Two types of cancers are included in this study. Colorectal cancer (CRC) is the third most common cancer and the second leading cause of cancer-related deaths. The American Cancer Society estimates that there will be about 149,500 new cases of CRC in the United States in 2023 and about 52,980 deaths from the disease. The high mortality rate of CRC is mainly attributed to the late diagnosis of the disease, which often leads to more advanced and difficult-to-treat stages. An early detection biomarker for CRC would benefit the patient and improve the outcome. ctDNA was proven to be a good biomarker for CRC, estimating minimal residual disease (MRD) and guiding therapy [21,26]. The second cancer is pancreatic cancer; it is one of the most lethal cancers due to late diagnosis. A total of 80% of patients present with late-stage disease and 11% with 5-year survival. However, stage 1 patients have an 80% survival rate [27]. An early detection biomarker can make a significant difference in disease outcome. 

We found that ctDNA is detectable in 34% of CRC patients. Over two-thirds of CRC patients had at least one mutation within the target regions of our panel. This can be improved by adding new regions and selecting other genes. However, we opted for practicality and kept a limited number of regions. This panel can help to monitor a significant portion of CRC patients without special modifications, thus achieving the main goal of its design.

Only 11% of those individuals were positive for ctDNA that were identified in the somatic mutation analysis. This is a poor result when considering that only half of the pancreas cancer patients have a mutation in the frequent regions selected. Our results show the success of the panel design in colon cancer cases for the same regions. Other studies proved a similar lower rate of detection in pancreas cancer [2]. For that, we conclude that pancreas cancer sheds lower amounts of ctDNA, if any. Our results point to differences in ctDNA among different types of cancers, thus negating the use of ctDNA as a universal biomarker for all types of malignancies. ctDNA analysis is outperformed in pancreas cancer by exosomes; thus, it may be better to focus on alternative analysis in this type of cancer [28].

A disappearance of ctDNA below detection levels is no guarantee that tumor relapse or metastasis will not happen. Two out of eight colon cancer patients (25%) with detectable ctDNA levels became ctDNA-negative sometime after therapeutical intervention. However, a relapse of tumor occurred later along with an increase in ctDNA. Interestingly, in one case, ctDNA returned a positive result 2 months before any other test could point to a tumor relapse or metastasis (Table 2—case study). Thus, postsurgery negative results are not a guarantee for a tumor-free recovery. And continuous ctDNA surveillance might be a better postsurgery surveillance strategy.

The goal from testing our approach was to decrease the costs of the test method while keeping acceptable sensitivity. Our savings came from reducing the costs of primer design and calibration and UMI lab and bioinformatics, and from using underutilized sequencing capacity on Illumina flowcells. With an average of 800,000 reads per sample for a full panel, the costs of one ctDNA test with dedicated flowcell capacity would be less than USD 60, excluding the costs of staff labor. The value of a ctDNA test is the positive result. The strategy to use this panel would be to test for ctDNA in patients with a primary tumor. If tested positive with the ctDNA panel, then the patient is a candidate for this analysis. With a possible pricing of USD 300, the final costs of the ctDNA strategy can drop from USD 8500 [11] to USD 5100. We already proved the method useful in over 30% of colon cancer patients. That would amount to a significant savings value if this strategy were applied for all colon cancer patients, in addition to the benefit to the patients from using precise tumor detection. The remaining 70% would benefit from personalized ctDNA panels or other monitoring techniques. 

The sensitivity of the method is another argument we make. Some methods of ctDNA analysis claim detection of mutant allele frequency (MAF) as low as 0.02%, such as CAPP-seq [24]. Our method can detect MAF ~0.01% (two reads for a coverage of 20,000). Our results clearly show an increased variability with lower concentrations of ctDNA (Figure 1b). Additionally, we were not able to link detection of low levels of ctDNA to the tumor status. Most of the patients (59%) had detectable ctDNA levels; however, the specificity of such positivity corresponded to many stable clinical cases. The more reasonable 0.5% cutoff is easily correlated to significant clinical changes. ctDNA levels for samples of over initial 0.5% ctDNA decreased in successfully treated patients and increased with tumor regrowth or metastasis. This leads us to suspect that tumors that shed low levels of ctDNA are less reliably monitorable by ctDNA methods in general. Although different methods have different sensitivity values, we expect that we will reach a level of disconnect between ctDNA and clinical significance above the level of our ability of detection. The higher sensitivity will not mean better detection. We suggest setting this value at 0.5% for ctDNA in colon cancer. Further studies might reveal a clearer limit of ctDNA usefulness.

ctDNA panels can be used to a significant degree to follow up with colon cancer patients after surgery and therapy. We recommend frequent monitoring of those patients, since the ease of use, impact, and predictive value of a positive finding are relatively high. To help manage the costs of monitoring, we suggest using simplified semiquantitative ctDNA panels like we present in this manuscript. Although these panels are not perfect, the economics and sensitivity justify monitoring patients who had positive ctDNA at surgery. More specific approaches for confirmation of positive results might be considered as a second line. In contrast, pancreatic cancer remains a challenging disease and we see little benefit in using this approach to measure ctDNA in this disease.

## 5. Conclusions

We succeeded in designing and testing a ctDNA panel targeting the most frequently mutated regions in colon cancers. This panel could be a building block for more advanced panels to follow up colon cancer or other cancers. The open-source panel can save significant costs for over 30% of colon cancer patients, and the benefit-to-cost remains to be evaluated in each type of cancer.

## Figures and Tables

**Table 1:** A summary of ctDNA detected in one point at least (>0.3% of total cfDNA) (ND = not detected).

ID	Cancer	Detected ctDNA	ctDNA Concordant with Clinical Data
sample1	Colon	ND	-
sample2	Colon	Detected	Concordant with clinical data
sample3	Colon	Detected	Concordant with clinical data
sample5	Colon	Detected	Concordant with clinical data
sample7	Colon	Detected	Concordant with clinical data
sample8	Colon	ND	-
sample9	Colon	ND	-
sample10	Colon	Detected	Concordant with clinical data
sample11	Colon	Detected	Not concordant with clinical data
sample12	Colon	ND	-
sample13	Colon	Detected	Concordant with clinical data
sample15	Pancreas	ND	-
sample16	Pancreas	ND	-
sample17	Pancreas	Detected	Concordant with clinical data
sample18	Pancreas	ND	-
sample19	Pancreas	ND	-

**Table 2:** Patient/sample2 case: the patient had detectable ctDNA from 1st to 3rd time-point. ctDNA was not detectable at time-point 4 but returned to high levels 2 months before imaging revealed disease progression.

Follow-Up	PIK3CA (c.1624G>A)	TP53 (c.403T>C)	Date	Clinical
1	1.7%	0.6%	April 2019	Surgery of primary tumor
2	0.8%	0.3% (ND)	May 2019	Metastasis surgery
3	0.9%	0.7%	August 2020	2 metastasis surgeries
4	ND	ND	October 2020	2 months after surgery/therapy
5	1.7%	1.2%	January 2022	Possible relapse but not identified in imaging (December 2022) Tumor identified via imaging (May 2022)

## Data Availability

Raw data are available upon reasonable request.

## Supplementary Materials

The following supporting information can be downloaded at: https://www.mdpi.com/article/10.3390/life13122274/s1; Figure S1: Useful correlations in the study cohort; Figure S2: Optimization of the panel; Table S1: The primer design; Table S2: Detailed table of the follow-up results.
