# Supplementary material for: Basic ctDNA Panel Promises Affordable Clinical Validity in Colon Cancer Patients but Not in Pancreas Cancer Patients
Source: Life (Basel). 2023 Nov 28;13(12):2274. doi: 10.3390/life13122274 (PMC10744654; doi:10.3390/life13122274)
Supplement: Supplementary file 1 [file life-13-02274-s001.zip › Supplementary_figures.pptx]

## Slide 1
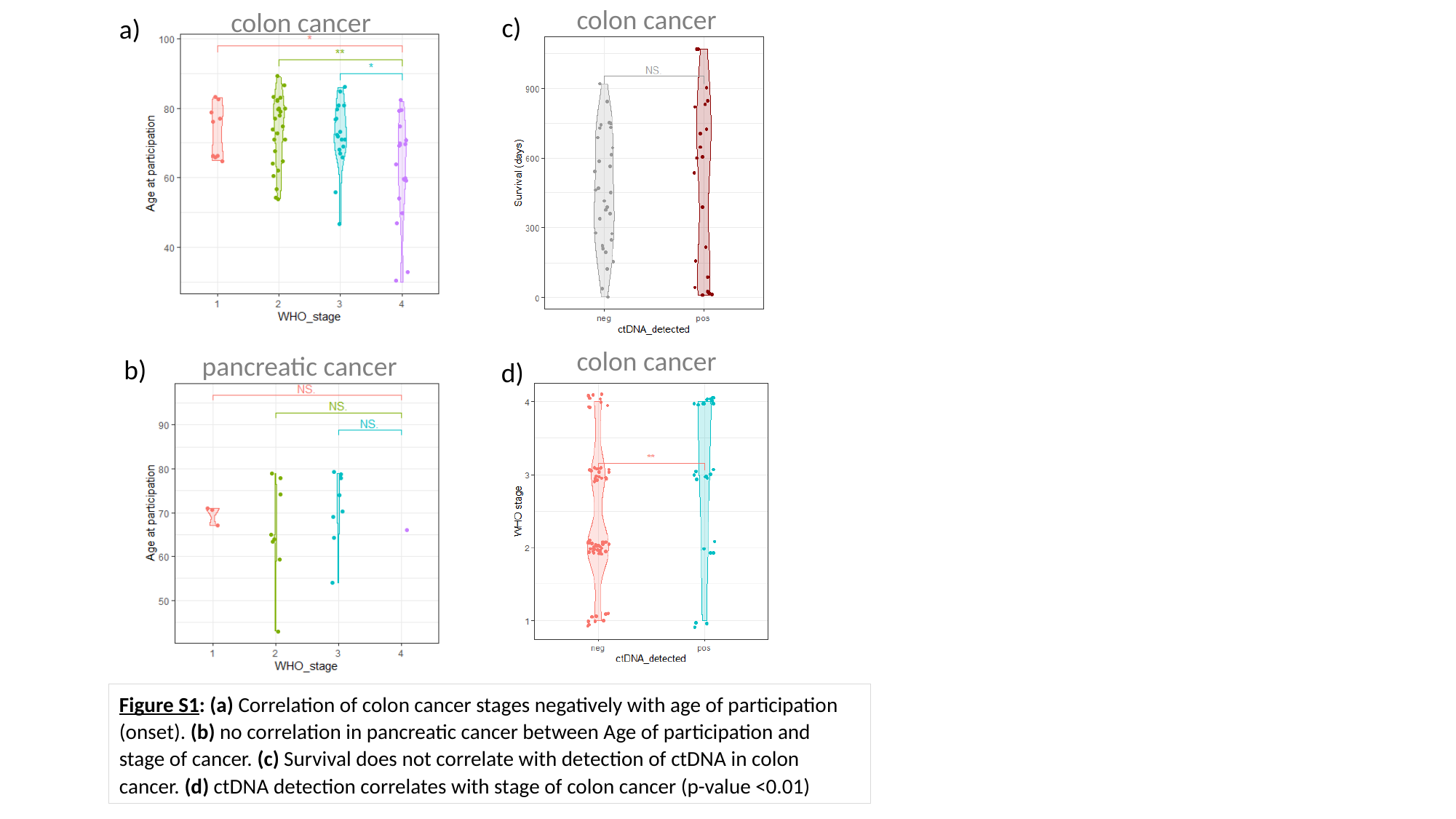

colon cancer
colon cancer
c)
a)
colon cancer
pancreatic cancer
b)
d)
Figure S1: (a) Correlation of colon cancer stages negatively with age of participation (onset). (b) no correlation in pancreatic cancer between Age of participation and stage of cancer. (c) Survival does not correlate with detection of ctDNA in colon cancer. (d) ctDNA detection correlates with stage of colon cancer (p-value <0.01)

## Slide 2
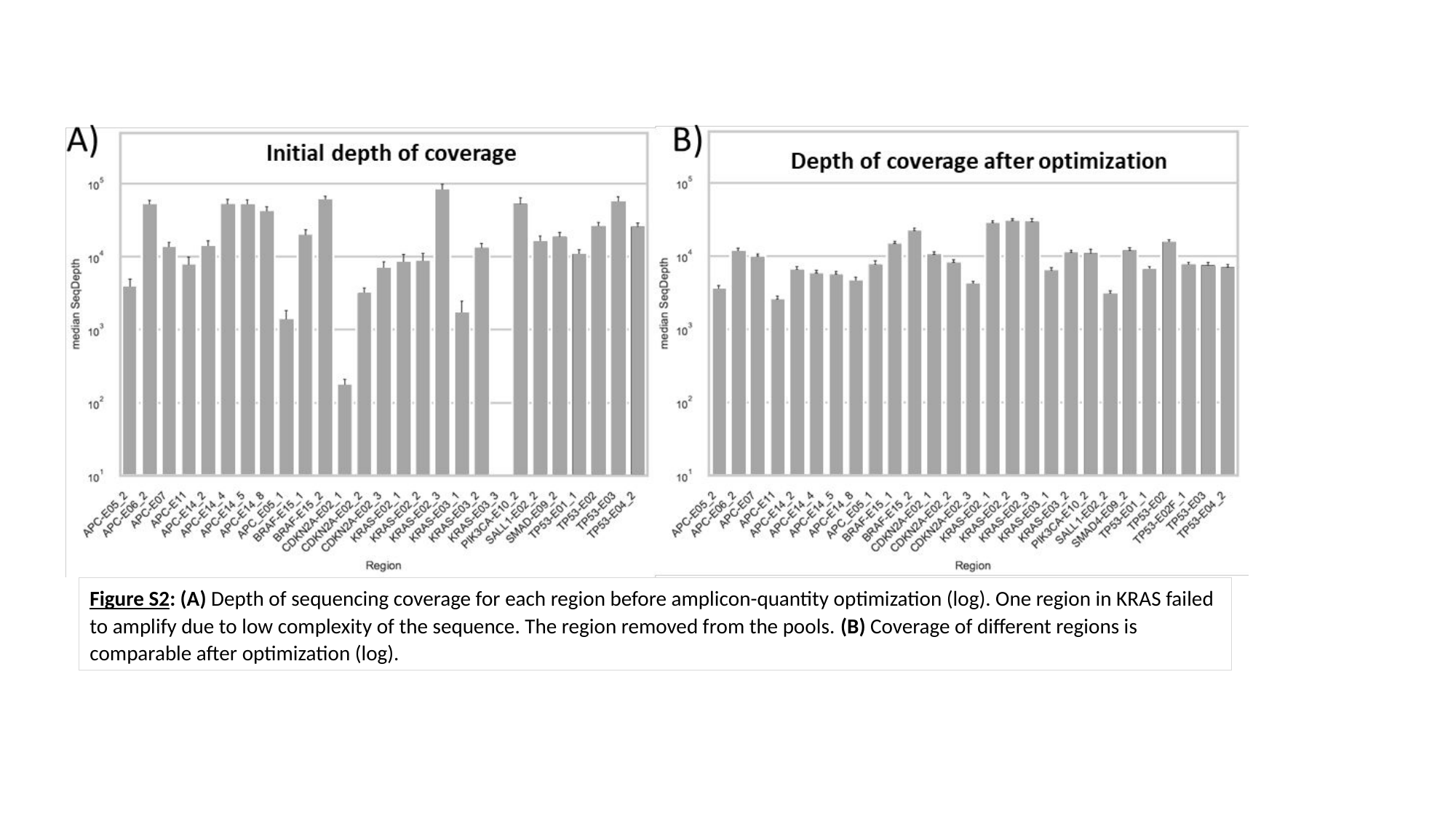

A)
B)
Initial depth of coverage
Depth of coverage after optimization
Figure S2: (A) Depth of sequencing coverage for each region before amplicon-quantity optimization (log). One region in KRAS failed to amplify due to low complexity of the sequence. The region removed from the pools. (B) Coverage of different regions is comparable after optimization (log).
